# Line-Drawn Scenes Provide Sufficient Information for Discrimination of Threat and Mere Negativity

**DOI:** 10.1177/2041669518755806

**Published:** 2018-02-12

**Authors:** Jasmine Boshyan, Lisa Feldman Barrett, Nicole Betz, Reginald B. Adams, Kestutis Kveraga

**Affiliations:** Athinoula A. Martinos Center for Biomedical Imaging, Massachusetts General Hospital, Charlestown, MA, USA; Department of Radiology, Harvard Medical School, Boston, MA, USA; Athinoula A. Martinos Center for Biomedical Imaging, Massachusetts General Hospital, Charlestown, MA, USA; Department of Psychology, Northeastern University, Boston, MA, USA; Department of Psychiatry, Massachusetts General Hospital, Charlestown, MA, USA; Department of Psychology, Northeastern University, Boston, MA, USA; Department of Psychology, The 8082 Pennsylvania State University, University Park, PA, USA; Athinoula A. Martinos Center for Biomedical Imaging, Massachusetts General Hospital, Charlestown, MA, USA; Department of Radiology, Harvard Medical School, Boston, MA, USA

**Keywords:** threat perception, line drawings, affect, emotion

## Abstract

Previous work using color photographic scenes has shown that human observers are keenly sensitive to different types of threatening and negative stimuli and reliably classify them by the presence, and spatial and temporal directions of threat. To test whether such distinctions can be extracted from impoverished visual information, we used 500 line drawings made by hand-tracing the original set of photographic scenes. Sixty participants rated the scenes on spatial and temporal dimensions of threat. Based on these ratings, trend analysis revealed five scene categories that were comparable to those identified for the matching color photographic scenes. Another 61 participants were randomly assigned to rate the valence or arousal evoked by the line drawings. The line drawings perceived to be the most negative were also perceived to be the most arousing, replicating the finding for color photographic scenes. We demonstrate here that humans are very sensitive to the spatial and temporal directions of threat even when they must extract this information from simple line drawings, and rate the line drawings very similarly to matched color photographs. The set of 500 hand-traced line-drawing scenes has been made freely available to the research community: http://www.kveragalab.org/threat.html.

## Introduction

Our visual system has been honed by the need for survival. From a continuous flood of visual information, our eyes and brain quickly extract meaning and scan for signs of danger, as rapid recognition of threat promotes survival (LeDoux, 2012). Previous studies have shown that stimuli are automatically represented in terms of their affective valence (Barrett, 2006; Duckworth, Bargh, Garcia, & Chaiken, 2002). But dimensional approaches to affective perception that map the valence and arousal of stimuli do not distinguish between unpleasant images that are perceived as aversive and threatening and those that are not (for a review, see Barrett & Bliss-Moreau, 2009; Barrett & Russell, 1999; Larsen & Diener, 1992; Russell, 1980; Watson & Tellegen, 1985). We have shown that not all negative stimuli are threatening, and that merely negative, but not threatening, stimuli evoke qualitatively different brain activation patterns (Kveraga et al., 2015). For example, imminent threat and accident scenes are both unpleasant situations, but responses to these types of images are quite different. While detecting imminent danger is critical for immediate survival, identifying appetitive stimuli and learning to avoid potential future threats are essential for long-term wellness and survival. Indeed, a recent study has confirmed that human observers exhibit “morbid curiosity” for socially negative, but not threatening, images (Oosterwijk, 2017).

It has been proposed that responses to some types of threat stimuli such as spiders, snakes, and angry faces may be innate and shaped by evolution (Brosch, Pourtois, & Sander, 2010; Isbell, 2006; Le et al., 2013; New, Cosmides, & Tooby, 2007; Seligman, 1971). For example, it has been demonstrated that spiders and snakes are detected more rapidly than mushrooms and flowers (Öhman, Flykt, & Esteves, 2001) and angry faces are detected faster than neutral faces and happy faces (Eastwood, Smilek, & Merikle, 2001; Hansen & Hansen, 1988; Horstmann, 2007; Tipples, Atkinson, & Young, 2002). However, the latter effect is clearly seen in schematic, line-drawn faces (Öhman, Soares, Juth, Lindström, & Esteves, 2012), but not necessarily in photographic faces because of the higher salience, or “vividness”, of happy, smiling faces (e.g., Becker, Anderson, Mortensen, Neufeld, & Neel, 2011; Becker & Srinivasan, 2014). Similar results were reported for children (LoBue, 2009, 2010a; LoBue & DeLoache, 2008) and infants (LoBue & DeLoache, 2010). Threatening faces are processed faster than are other facial expressions (Schupp et al., 2004). In addition, saccadic eye movements orient more quickly to images of threatening compared to neutral faces and body postures (Bannerman, Milders, de Gelder, & Sahraie, 2009) as well as towards emotional compared to neutral scenes (Nummenmaa, Hyona, & Calvo, 2009). While these studies support the view that spiders, snakes, and angry faces belong to a special class of stimuli that are perceptually prioritized due to their importance for survival, adults have been also shown to quickly detect modern threats, such as guns, knives, and syringes (e.g., Blanchette, 2006). Using the same stimuli, LoBue (2010b) reported that children only detected the syringes particularly quickly. These findings suggest that humans learn to detect novel threatening stimuli efficiently as a result of negative experiences. Together the research suggests that learning plays a vital role in threat perception. Humans and other primates not only have biases for the rapid detection of evolutionarily ancient threats such as snakes and spiders (Isbell, 2006) but also have the flexibility to learn to efficiently detect new threats that are specific to our environments (LoBue, Rakison, & DeLoache, 2010).

**Figure 1.**
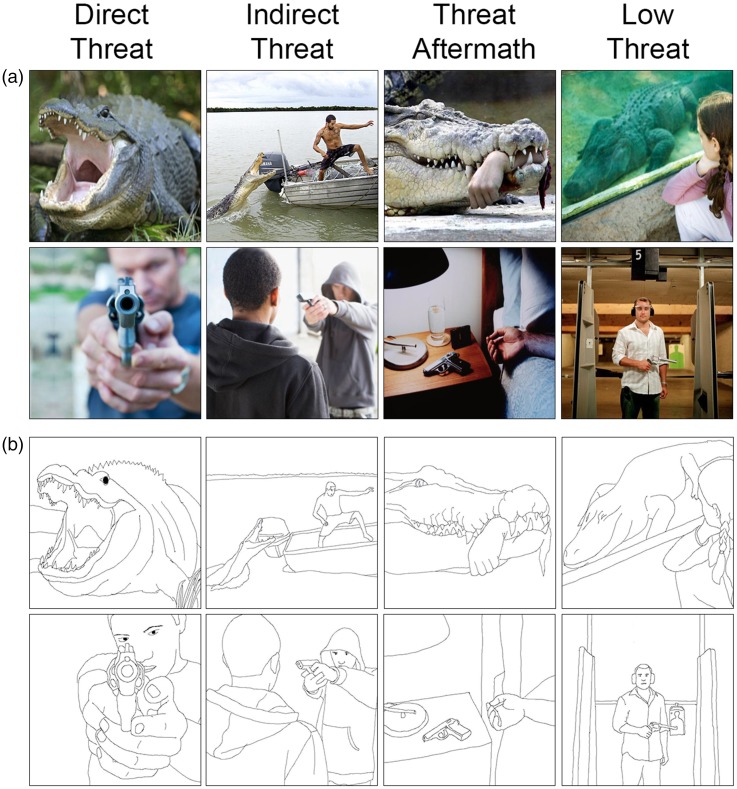
(a) Examples of color photographic scene stimuli used in Kveraga et al. (2015) depicting human and animal Direct Threat, Indirect Threat, Threat Aftermath, and Low Threat situations. (b) Line-drawing scene stimuli used in the current study.

**Figure 2.**
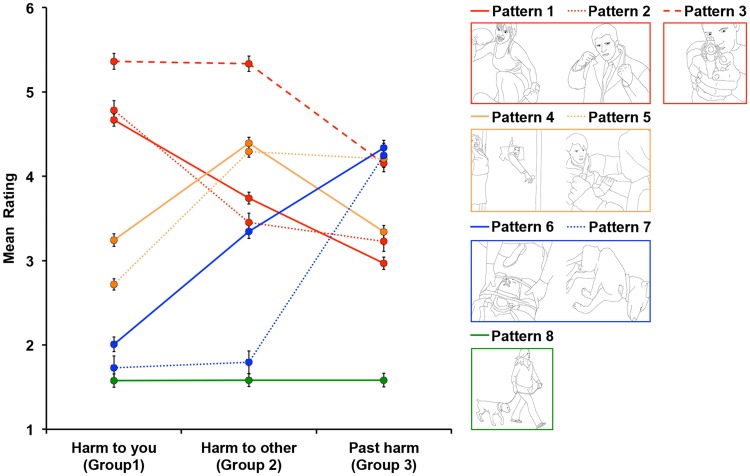
Eight unique patterns capturing 500 images in our stimulus set based on trend analysis. Patterns 1 (*n* = 85), 2 (*n* = 34), and 3 (*n* = 51) all captured images that depicted scenes posing high threat to the observer, receiving high ratings from *Harm to you* group. Both Patterns 4 (*n* = 80) and 5 (*n* = 97) captured images depicting scenes posing high threat to someone else, receiving highest ratings from *Harm to other* group. Patterns 6 (*n* = 59) and 7 (*n* = 23) both captured images depicting scenes where harm (physical, psychological, or both) has already happened, receiving highest ratings from *Past harm* group. Finally, Pattern 8 (*n* = 71) captured images depicting low level of threat either to the observer or someone else that were rated equally low by all three groups.

In a recent study focusing on how humans perceive and process negative stimuli (Kveraga et al., 2015), we presented color photographic scenes, including both natural threats (e.g., snakes, spiders, and carnivorans) as well as manmade threats (e.g., humans pointing guns and knives) classified a priori into four categories: Direct Threat, Indirect Threat, Threat Aftermath, and Low Threat situations (Figure 1(a)). We demonstrated that humans discriminate these four types of stimuli quickly and accurately. The images containing direct, immediate threats were recognized most quickly, while Threat Aftermath (merely negative) scene images without imminent threats were processed much more slowly, with responses lagging behind even Low Threat images. In addition, we found that threatening and merely negative scene images activated different, though somewhat overlapping, networks of brain regions. Specifically, the scene images depicting threat situations differentially activated the amygdalae, periaqueductal gray, and orbitofrontal cortex, while merely negative, Threat Aftermath scenes evoked stronger activity in the parahippocampal, retrosplenial, medial and lateral prefrontal, and lateral temporal cortices (Kveraga et al., 2015; Figure 2).

Line drawings have been long used as a tool for studying object and scene perception because they are much easier to systematically manipulate (e.g., altering eye gaze, body poses, presence or absence of a certain object and so on) compared to photographic images (Biederman, Mezzanotte, & Rabinowitz, 1982; Kveraga, Boshyan, & Bar, 2007; Palmer, 1975; Walther, Chai, Caddigan, Beck, & Li, 2011). For example, in their seminal studies, Biederman et al. (1982) and Palmer (1975) demonstrated the effects of context on object recognition by manipulating line-drawing scenes. In another study, Kveraga et al. (2007) used line drawings to investigate the contribution of the magnocellular and parvocellular visual pathways to object recognition. More recently, Walther et al. (2011) reported that despite the marked difference in nonaffective scene statistics, they were able to decode scene category from fMRI data for nonaffective line dawings just as well as from activity for color photographs, in primary visual cortex through parahippocampal place area and retrosplenial cortex. Even more remarkably, in these regions, error patterns for decoding from line drawings were very similar to those obtained using color photographs.

Several general and specialized visual stimulus sets have been developed to facilitate behavioral research on emotion, such as the International Affective Picture System (IAPS; Lang, Bradley, & Cuthbert, 2005), the Geneva Affective Picture Database (GAPED; Dan-Glauser & Scherer, 2011), and the Open Affective Standardized Image Set (OASIS; Kurdi, Lozano, & Banaji, 2017). IAPS images, for example, have been used in several thousand research studies since they were first made available to the research community. However, IAPS images are subject to copyright restrictions that prohibit their usage in online research studies. Because of this, researchers conducting online studies were forced to create visual stimuli in an ad hoc manner, which was time-consuming, inefficient, and limited the comparability and generalizability of research findings. The OASIS database was specifically developed to address this problem by providing an open-access standardized stimulus set containing affective images with corresponding normative affective ratings, which similarly to the IAPS included a broad spectrum of themes.

Even though it has been previously demonstrated that line-drawing images are a useful tool for studying objects and scenes, no line-drawn stimuli set of affective scenes exists to date. The goals of the present study were twofold: (a) to test whether distinctions in affective scene context that allow humans to discriminate different dimensions of threat and negativity can be extracted from very basic, impoverished visual information such as line drawings and (b) to compile a set of open-access images varying in their threat value that differ little in low-level features and are easier to manipulate compared to photographic images to facilitate studies of visual threat perception in neurotypical, as well as clinical, populations.

## Method

### Participants

#### Study 1

Sixty participants were recruited from Northeastern University and were rewarded with course credit for their participation. Eight participants were removed from the analyses due to poor performance (see Supplemental Tables 1 and 2 for details). The remaining 52 participants (27 males and 25 females) had a mean age of 19.49 (*SD* = 1.50). Participants reported their ethnicity as European American (*n* = 31), Asian (*n* = 15), and African American (*n* = 1). Five participants chose not to report their race.

#### Study 2

Sixty-one participants were recruited from the Pennsylvania State University and were rewarded with course credit for their participation. One participant was removed from the analyses due to poor performance. The remaining 60 participants (29 males and 31 females) had a mean age of 19.28 (*SD* = 1.22). Participants reported their ethnicity as European American (*n* = 34), Asian (*n* = 13), African American (*n* = 9), and Hispanic American (*n* = 2). Two participants chose not to report their race.

### Images

We previously collected a set of 509 color photographic images depicting negative and neutral situations from Internet searches. As reported in Kveraga et al. (2015), prior to collecting any data, we had classified these images into four categories based on our initial impressions: (a) negative, Direct Threat (from the viewpoint of the observer); (b) negative, Indirect Threat (predominantly to others); (c) negative, Threat Aftermath (a possible past threat, but not a threat any longer); and (d) neutral, Low Threat. We selected 500 out of these images to create line drawings by hand-tracing the main contours of agents (humans and animals), objects, and backgrounds. The line drawings were 700 × 700 pixels, with a black foreground and white background (e.g., see Figure 1b).

### Procedure

#### Study 1

Participants were invited to the laboratory where they first read and signed the consent form. They were then randomly assigned to one of three groups. Each group of participants only responded to one of the questions on a scale from 1 to 6 (from *none* to *extreme*). Five hundred line-drawing scene images were presented in a random order, and one of the following three questions was presented below each picture: (a) “How much harm might you be about to suffer if this was your view in this scene?” (*Harm to you* group), (b) “How much harm might someone else (not you) be about to suffer in this scene?” (*Harm to other* group), and (c) “How much harm might someone else (not you) have already suffered in this scene?” (*Past harm* group). Participants could view each stimulus as long as they wanted and enter their rating using keys 1 to 6 on the keyboard. The rating scale was placed below the image and the points of the scale were labeled as *None*, *Little*, *Some*, *Quite a lot*, *Even more*, and *Extreme*. Entering the rating replaced the current stimulus with the next one. In order to discourage participants from rushing through the ratings, the program would not move to the next image if the response was made sooner than 500 ms after stimulus presentation. Instead, the image would stay on the screen for at least 500 ms, and participants would have to repeat their response in order to proceed to the next image. Even though a 500 ms threshold was employed to discourage participants from speeding, we also collected reaction times (RT) to screen for participants who nevertheless speeded through the task or took an extraordinarily long time to respond, as compared to other participants. After rating all the images, participants were asked to fill out a demographic information form, debriefed, and thanked for their time. All procedures were approved by the Northeastern University Institutional Review Board (IRB#11-03-35).

#### Study 2

Participants were invited to the laboratory where they first read and signed the consent form. They were then randomly assigned to rate five hundred line-drawing scene images (presented in an individually randomized order) on only the valence dimension or only the arousal dimension using a 7-point Likert scale. We used image-focused instructions for valence and arousal ratings following the procedure described in Kurdi et al. (2017). Participants could view each stimulus as long as they wanted and enter their rating using keys 1 to 7 on the keyboard. The rating scale was placed below the image. For the valence dimension, the word “Valence” was displayed above the rating scale and the points of the scale were labeled as *Very positive*, *Moderately positive*, *Somewhat positive*, *Neutral*, *Somewhat negative*, *Moderately negative*, and *Very negative*. For the arousal dimension, the word “Arousal” was displayed above the rating scale and the points of the scale were labeled as *Very low*, *Moderately low*, *Somewhat low*, *Neither low nor high*, *Somewhat high*, *Moderately high*, and *Very high*. Entering the rating replaced the current stimulus with the next one. After rating all the images, participants were asked to fill out a demographic information form, debriefed, and thanked for their time. All procedures were approved by the Pennsylvania State University Institutional Review Board (IRB#00005514).

## Results

### Study 1

The RT data were first examined to screen for evidence of speeding through the task. One-sample *t* tests comparing mean RT of each participant to the mean RT of their respective groups revealed that two participants were significantly faster as compared to other participants in their corresponding groups (both *p*s = .0001). Thus, we excluded their data from further analyses. In addition, we examined the RT distribution of each participant, removing any responses that were 2 standard deviations above the mean RT of corresponding participant (Ratcliff, 1993). This procedure resulted in the removal of 3.73% of the data. Next, we examined interrater reliability for each group. This analysis identified six additional participants whose responses were significantly different from the responses of other participants in their respective groups. The resulting mean interreliability across all three rating groups was .979 (*Harm to you* group: *n* = 18, .986; *Harm to other* group: *n* = 17, .983; *Past harm* group: *n* = 17, .969).^1^

#### Patterns

Means and standard deviations were calculated for each image as rated by each group. We then ran one-way ANOVAs on ratings from the three groups for each image to find the best fitting pattern for each image by fitting eight contrasts capturing different patterns (Figure 2).^2^ Based on the ratings across the three groups, we identified three patterns of images depicting scenes posing high threat to the observer (Direct Threat). Pattern 1 (*n* = 85) included images depicting scenes where the observer was the target of potential harm, but where potential harm to someone else was less, and already occurring harm was even less. Pattern 2 (*n* = 34) included images depicting scenes where the observer was the target of potential harm with lower but equal potential harm to someone else and already occurring harm. Like Patterns 1 and 2, images captured by Pattern 3 (*n* = 51) received high ratings by *Harm to you* group. However, unlike the other two patterns, these images were rated equally high by *Harm to other* group, followed by lower ratings by *Past harm* group. While examining the content of the scenes in the Direct Threat category we did not see any obvious differences between the images fitting Patterns 1 and 2, whereas stimuli fitting Pattern 3 depicted scenes with deadly weapons pointing directly at the observer. It is worth noting that none of the images fitting Pattern 3 included any animals. In addition, participants in *Harm to you* group rated stimuli fitting Pattern 3 (*M* = 5.36, *SD* = .06) significantly higher as compared to images fitting both Pattern 1 (*M* = 4.67, *SD* = .05), *t* = 8.41, *p* < .001 and Pattern 2 (*M* = 4.78, *SD* = .06), *t* = 7.73, *p* < .001, with no difference between the latter two, *t* = 1.12, *p* = .265. Thus, we combined the stimuli fitting Patterns 1 and 2 into one Direct Threat category (*n* = 119), but separated images fitting Pattern 3 into a category we called Deadly Threat (*n* = 51).

Patterns 4 (*n* = 80) and 5 (*n* = 97) both captured images depicting scenes posing high threat to someone else (Indirect Threat). However, images captured by Pattern 4 received highest ratings by *Harm to other* group with equally lower ratings by *Harm to you* and *Past harm* groups, while images captured by Pattern 5 received equally high ratings by *Harm to other* and *Past harm* groups with lower ratings by *Harm to you* group. No differences in content were found between images fitting Patterns 4 and 5 and participants in *Harm to other* group rated stimuli fitting Pattern 4 (*M* = 4.39, *SD* = .73) and Pattern 5 (*M* = 4.29, *SD* = .61) equally, *t* = .97, *p* = .334. Hence, we combined the stimuli of these two patterns into one Indirect Threat category (*n* = 177).

Patterns 6 (*n* = 59) and 7 (*n* = 23) both captured images depicting scenes where harm (physical, psychological, or both) has already happened (Threat Aftermath). Images captured by Patterns 6 and 7 received highest ratings from *Past harm* group and lowest ratings from *Harm to you* group. However, *Harm to other* group rated images characterized by Pattern 6 higher than *Harm to you* group but lower than *Past harm* group, while they rated images captured by Pattern 7 equally low as *Harm to you* group. Visual examination of image content of scenes captured by Patterns 6 and 7 revealed that while both depicted images of obvious past harm, all images characterized by Pattern 7 depicted dead (or mortally injured) animals. However, because no statistically significant difference was found between the ratings of participants from *Past harm* group for images in Pattern 6 (*M* = 4.34, *SD* = .95) and Pattern 7 (*M* = 4.25, *SD* = .66), *t* = .42, *p* = .680, we combined these images into one Threat Aftermath category (*n* = 82).

Finally, Pattern 8 (*n* = 71) captured images depicting low level of threat either to the observer or someone else (Low Threat; e.g., an image of a woman walking a dog on a leash) that were rated equally low by all three groups.

#### Image categories

**Figure 3.**
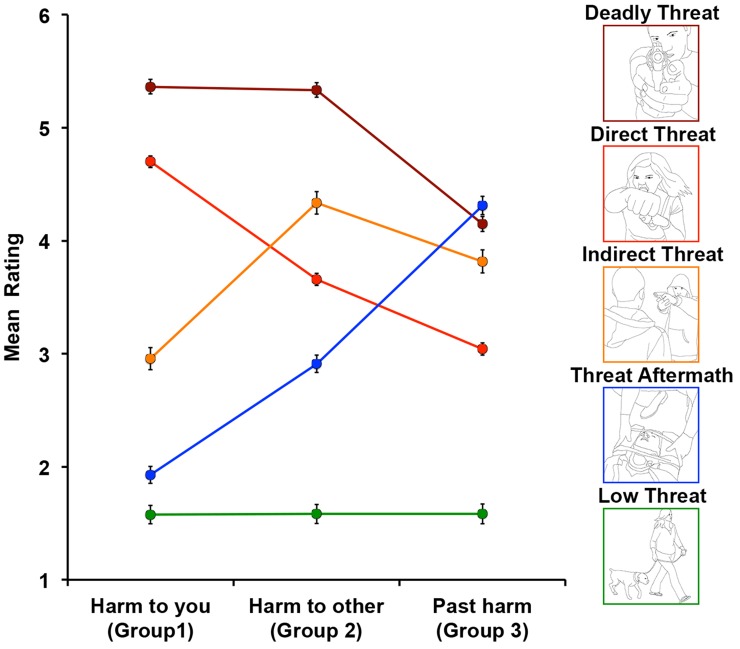
Five distinct image categories with corresponding prototypical examples.

Based on trend analyses we created five image categories (Figure 3). *Deadly Threat* category (*n* = 51) included images depicting scenes with deadly weapons pointing directly at the observer with equal potential harm to someone else, but lower possibility of harm already occurring. It is worth noting that none of the images in this category included any animals. *Direct Threat* category (*n* = 119) included images depicting scenes where the observer was the target of potential harm, but where potential harm to someone else was less, and already occurring harm was even less. *Indirect Threat* category (*n* = 177) included images depicting scenes with highest harm to someone else, lower possibility of harm already occurring, and the lowest potential harm to the observer. *Threat Aftermath* category (*n* = 82) included images depicting scenes where past harm (physical, psychological, or both) has already occurred, with lower potential harm to someone else, and lowest potential harm to the observer. Finally, *Low Threat* category (*n* = 71) included images depicting low level of harm to the observer or someone else, as well as low possibility of past harm already occurring.

#### Mean ratings

**Figure 4.**
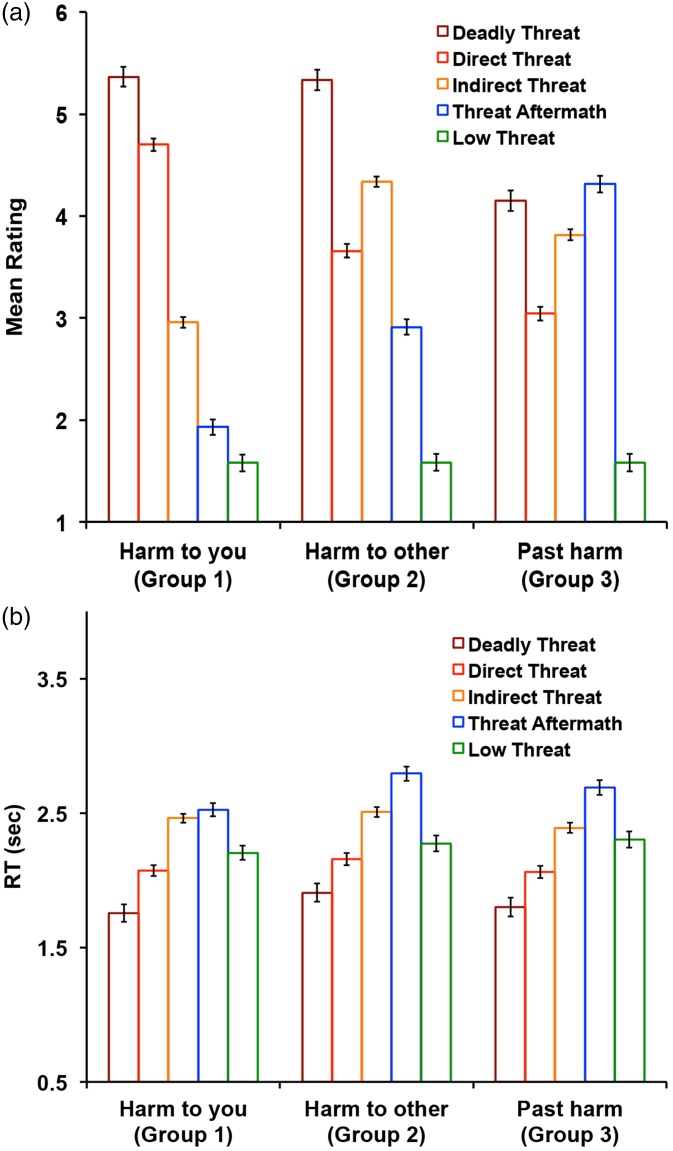
Study 1 results. (a) Results of the scene ratings. The three panels show the results from the three rating groups. Participants in Group 1 (left panel) were asked to rate all images in terms of potential harm to them personally; participants in Group 2 (middle panel) were asked to rate all images in terms of potential harm to someone else; participants in Group 3 (right panel) were asked to rate all images in terms of past harm, but no present harm. 500 images were presented in a randomized mixed sequence. (b) Results of the RT. The error bars indicate standard error of the mean.

To assess the statistical significance of the differences in the ratings, we ran a 3 (rating group) × 5 (image category) repeated-measures ANOVA. In order to account for eventual biases due to unbalanced numbers of stimuli in each image category, we used Type II calculation for obtaining sums of squares.The analysis revealed a significant main effect of rating group (*F*(2,990) = 122.48, *p* < .001, *η*^2^_*p*_ = .198) with *Harm to other* group having the highest overall ratings as compared to *Harm to you* and *Past harm* groups (both *p*s < .001), with no significant difference between the latter two (*Harm to you* group: *M*_difference_ = 3.31, *SE* = .03; *Past harm* group: *M*_difference_ = 3.38, *SE* = .04, *t*(998) = 2.27, *p* = .066). There was also a significant main effect of image category (*F*(4,495) = 253.31, *p* < .001, *η*^2^_*p*_ = .672). Except for a nonsignificant difference between Direct (*M* = 3.80, *SE* = .06) and Indirect Threat (*M* = 3.70, *SE* = .08) categories (*t*(294) = 1.29, *p* = .884), all image categories were significantly different from each other as indicated by post hoc pairwise comparisons with a Bonferroni correction (all *p*s < .001). This nonsignificant difference between Direct Threat and Indirect Threat image categories, collapsing across rating groups, was due to the difference in ratings for these images among all the three groups of participants. Specifically, while participants in *Harm to you* group rated Direct Threat images higher than Indirect Threat images, the opposite was true for the participants in *Harm to other* and *Past harm* groups. Thus, when collapsing across the three groups, the difference between Direct Threat and Indirect Threat image categories was nonsignificant. This is illustrated by a significant interaction between the image category and rating group (*F*(8,990) = 445.96, *p* < .001, *η*^2^_p_ = .783; Figure 4). *Harm to you* group had the highest ratings for the Deadly Threat image category compared to all other categories (all *p*s < .001), followed by Direct Threat (all *p*s < .001), Indirect Threat (all *p*s < .001), Threat Aftermath (all *p*s < .017), and Low Threat (all *p*s < .017) as indicated by pairwise comparisons with a Bonferroni correction. Similarly for *Harm to other* group, all pairwise comparisons with Bonferroni correction were significant (all *p*s < .001). Participants rated Deadly Threat images the highest followed by Indirect Threat, Direct Threat, Threat Aftermath, and Low Threat categories. For *Past harm* group, all pairwise comparisons with Bonferroni correction were significant (all *p*s < .043), except for the comparison between the Deadly Threat (*M* = 4.15, *SE* = .10) and Threat Aftermath image categories (*M* = 4.31, *SE* = .08; *t*(131) = 1.24, *p* = .908). Participants rated Threat Aftermath and Deadly Threat images the highest, followed by Indirect Threat, Direct Threat, and Low Threat categories.

#### Reaction times

By design, we enforced a 500-ms threshold to discourage participants from speeding through the images; thus, the shortest RT in our collected data was 501 ms. Even though the interpretation of our RT data is limited, we still examined any possible differences in RT between the rating groups as well as image categories by performing a 3 (rating group) × 5 (image category) repeated-measures ANOVA using Type II calculation for obtaining sums of squares. The analysis revealed a significant main effect of image category (*F*(4,495) = 50.39, *p* < .001, *η*^2^_*p*_ = .289). Except for no statistically significant difference between the Direct Threat (*M* = 2.10, *SE* = .04) and Low Threat (*M* = 2.26, *SE* = .05) categories (*t*(188) = 2.72, *p* = .064), all image categories were significantly different from each other as indicated by post hoc pairwise comparisons with a Bonferroni correction (all *p*s < .006). Deadly Threat images were rated most quickly, followed by Direct Threat and Low Threat images, Indirect Threat, and finally Threat Aftermath images, which had the longest RT. There was also a significant main effect of rating group (*F*(2,990) = 14.78, *p* < .001, *η*^2^_*p*_ = .029), with *Harm to other* group having the longest overall RTs as compared to *Harm to you* and *Past harm* groups (both *p*s < .001), with no significant difference between the latter two (*Harm to you* group: *M*_difference_ = 2.20, *SE* = .02; *Past harm* group: *M*_difference_ = 2.25, *SE* = .03; *t*(998) = 1.88, *p* = .182). Finally, there was a significant interaction between the image category and rating group (*F*(8,990) = 3.04, *p* = .002, *η^2^_p_* = .024; Figure 4). *Harm to you* group provided fastest responses to the Deadly Threat image category (all *p*s < .001), followed by equally fast responses to Direct Threat and Low Threat images (all *p*s < .001, except for no statistically significant difference between Direct Threat (*M* = 2.07, *SE* = .04) and Low Threat images (*M* = 2.20, *SE* = .05), *t*(188) = 1.94, *p* = .426), and longest RT for Indirect Threat and Threat Aftermath images—all *p*s < .001, except for no statistically significant difference between Indirect Threat (*M* = 2.46, *SE* = .03) and Threat Aftermath (*M* = 2.53, *SE* = .05), *t*(257) = 1.05, *p* = .968—as indicated by pairwise comparisons with a Bonferroni correction. For *Harm to other* group, all pairwise comparisons with Bonferroni correction were significant (all *p*s < .023) except for no statistically significant difference between the Direct Threat (*M* = 2.16, *SE* = .05) and Low Threat (*M* = 2.27, *SE* = .06; *t*(188) = 1.59, *p* = .695) categories. Participants rated Deadly Threat images the fastest, followed by equally fast Direct and Low Threat images, followed by Indirect Threat and finally Threat Aftermath images with longest RTs. Similarly for *Past harm* group, all pairwise comparisons with a Bonferroni correction were significant (all *p*s < .020), except for the comparison between the Indirect Threat (*M* = 2.39, *SE* = .04) and Low Threat (*M* = 2.30, *SE* = .06) image categories *t*(246) = 1.26, *p* = .907. Participants again rated the Deadly Threat images the fastest followed by Direct Threat, equally slower Indirect Threat and Low Threat images and finally the slowest Threat Aftermath images.

### Study 2

Prior to analyses, valence ratings were reverse coded such that lower ratings would correspond to negative valence, while higher ratings would correspond to positive valence. Interrater reliability for both valence and arousal ratings was excellent (.943 and .959, respectively).^3^

**Figure 5.**
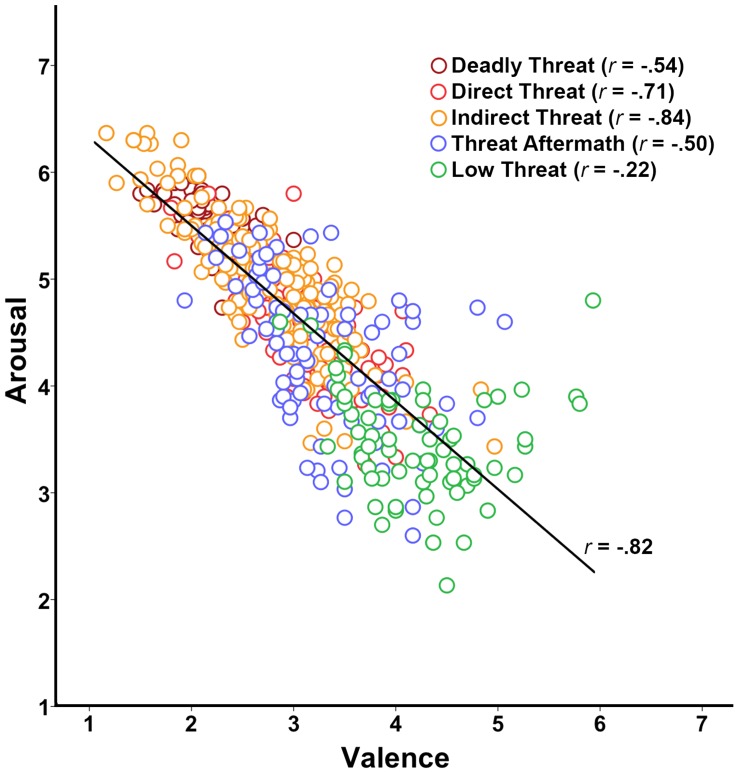
Study 2 results. The relationship between valence and arousal ratings, with valence (measured on a 1–7 Likert scale) on the *x*-axis and arousal (also measured on a 1–7 Likert scale) on the *y*-axis. The colors correspond to image categories.

The relationship between valence and arousal ratings is shown in Figure 5. Overall, there was a strong negative linear relationship between valence and arousal ratings, Pearson’s *r* = −.816, *p* < .001. Low Threat images had the highest mean valence rating (*M* = 4.21, *SE* = .07), followed by Threat Aftermath images (*M* = 3.30, *SE* = .06), Direct Threat images (*M* = 3.10, *SE* = .05), Indirect Threat images (*M* = 2.76, *SD* = .04), and Deadly Threat images (*M* = 2.14, *SD* = .08). On the arousal dimension, Deadly Threat images were rated as most highly arousing (*M* = 5.57, *SE* = .08), followed by Indirect Threat images (*M* = 4.99, *SE* = .04), Direct Threat images (*M* = 4.57, *SE* = .05), Threat Aftermath images (*M* = 4.28, *SE* = .06), and Low Threat images (*M* = 3.48, *SE* = .07).

### Comparison of Line-Drawn and Color Photographic Scenes

In order to validate our findings, we also reanalyzed the data collected and reported previously (Kveraga et al., 2015) for the color photographic images using the five image categories described above. In the previous study, 27 participants were randomly assigned to rate all color photographic images answering one of three questions: (a) How much harm might you be about to suffer in this scene if this was your view of the scene?; (b) How much harm might someone (not you) be about to suffer in this scene?; and (c) How much harm might someone (not you) have already suffered in this scene? Another 33 participants were asked to rate all the color photographic images on valence and arousal dimensions using 6-point Likert scales (for details see Kveraga et al., 2015).

#### Study 1

**Figure 6.**
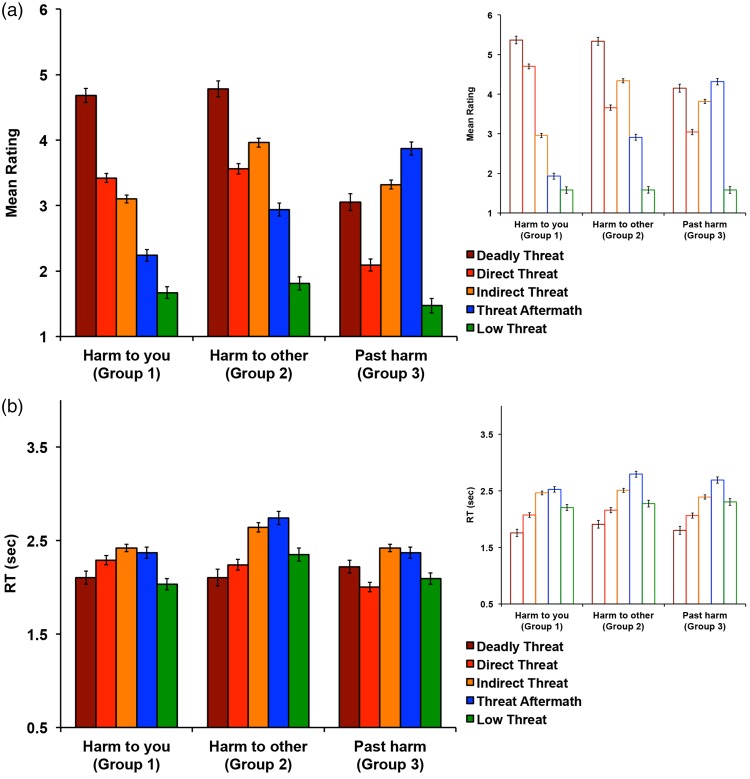
Study 1 validation results. (a) Results of the color photographic scene ratings (left). The three panels show the results from the three rating groups. Participants in Group 1 (left panel) were asked to rate all images in terms of potential harm to them personally; participants in Group 2 (middle panel) were asked to rate all images in terms of potential harm to someone else; participants in Group 3 (right panel) were asked to rate all images in terms of past harm, but no present harm. 500 images were presented in a randomized mixed sequence. Results of the line drawings of the matching scenes (Study 1) are presented on the right for the comparison. (b) Results of the RT. The error bars indicate standard error of the mean.

A 3 (rating group) × 5 (image category) repeated-measures ANOVA (using Type II calculation for obtaining sums of squares) on color photographic scenes revealed similar pattern of results as reported for the line-drawing images (Figure 6). Descriptive statistics and results summary are presented in Table 1.

**Table 1.** Results Summary Tables (a) and Descriptive Statistics (b) for the 3(Rating Group) × 5(Image Category) Repeated Measures ANOVA for Line Drawings (Study 1) and Matching Color Photographic Images (Validation Analysis for Study 1 Ran on Data Previously Reported in Kveraga et al., 2015).

**(a)**	Line drawings						Color photographs				
		*df*	*F*	Sig.	η^2^			*df*	*F*	Sig.	η^2^
Rating	Group	2,990	122.48	<.001	.198	Rating	Group	2,990	159.72	<.001	.244
	Category	4,495	253.31	<.001	.672		Category	4,495	114.95	<.001	.482
	Group × Category	8,990	445.96	<.001	.783		Group × Category	8,990	114.62	<.001	.481
Reaction times	Group	2,990	14.82	<.001	.029	Reaction times	Group	2,990	32.81	<.001	.062
	Category	4,495	50.39	<.001	.289		Category	4,495	20.66	<.001	.143
	Group × Category	8,990	3.04	.002	.024		Group × Category	8,990	6.37	<.001	.049

**(b)**											
	Line drawings						Color photographs				
		Rating		Reaction times				Ratin g		Reaction times	
	Group	*M*	*SE*	*M*	*SE*		Group	*M*	*SE*	*M*	*SE*
	Harm to you	3.31	0.03	2.20	0.02		Harm to you	3.02	0.04	2.24	0.02
	Harm to other	3.56	0.04	2.33	0.02		Harm to other	3.45	0.04	2.41	0.03
	Past harm	3.38	0.04	2.25	0.03		Past harm	2.76	0.05	2.22	0.03
	**Category**						**Category**				
	Deadly Threat	4.95	0.09	1.82	0.06		Deadly Threat	4.17	0.10	2.14	0.06
	Direct Threat	3.80	0.06	2.10	0.04		Direct Threat	3.02	0.07	2.18	0.04
	Indirect Threat	3.70	0.05	2.45	0.03		Indirect Threat	3.46	0.05	2.50	0.03
	Threat Aftermath	3.05	0.07	2.67	0.04		Threat Aftermath	3.01	0.08	2.49	0.05
	Low Threat	1.58	0.08	2.26	0.05		Low Threat	1.65	0.08	2.15	0.05
**Group**	**Category**					**Group**	**Category**				
Deadly Threat	Harm to you	5.36	0.10	1.76	0.06	Deadly Threat	Harm to you	4.68	0.11	2.10	0.07
	Harm to other	5.33	0.10	1.91	0.07		Harm to other	4.78	0.12	2.10	0.09
	Past harm	4.15	0.10	1.80	0.07		Past harm	3.05	0.13	2.22	0.07
Direct Threat	Harm to you	4.70	0.06	2.07	0.04	Direct Threat	Harm to you	3.42	0.07	2.29	0.05
	Harm to other	3.66	0.07	2.16	0.05		Harm to other	3.56	0.08	2.24	0.06
	Past harm	3.04	0.07	2.06	0.05		Past harm	2.09	0.09	2.00	0.05
Indirect Threat	Harm to you	2.96	0.05	2.46	0.03	Indirect Threat	Harm to you	3.10	0.06	2.42	0.04
	Harm to other	4.34	0.05	2.51	0.04		Harm to other	3.96	0.07	2.64	0.05
	Past harm	3.82	0.06	2.39	0.04		Past harm	3.32	0.07	2.42	0.04
Threat Aftermath	Harm to you	1.93	0.08	2.53	0.05	Threat Aftermath	Harm to you	2.24	0.09	2.37	0.06
	Harm to other	2.91	0.08	2.80	0.05		Harm to other	2.94	0.10	2.74	0.07
	Past harm	4.31	0.08	2.69	0.06		Past harm	3.87	0.10	2.37	0.06
Low Threat	Harm to you	1.58	0.08	2.20	0.05	Low Threat	Harm to you	1.67	0.09	2.03	0.06
	Harm to other	1.58	0.08	2.27	0.06		Harm to other	1.81	0.10	2.35	0.07
	Past harm	1.58	0.09	2.30	0.06		Past harm	1.47	0.11	2.09	0.06

#### Study 2

**Figure 7.**
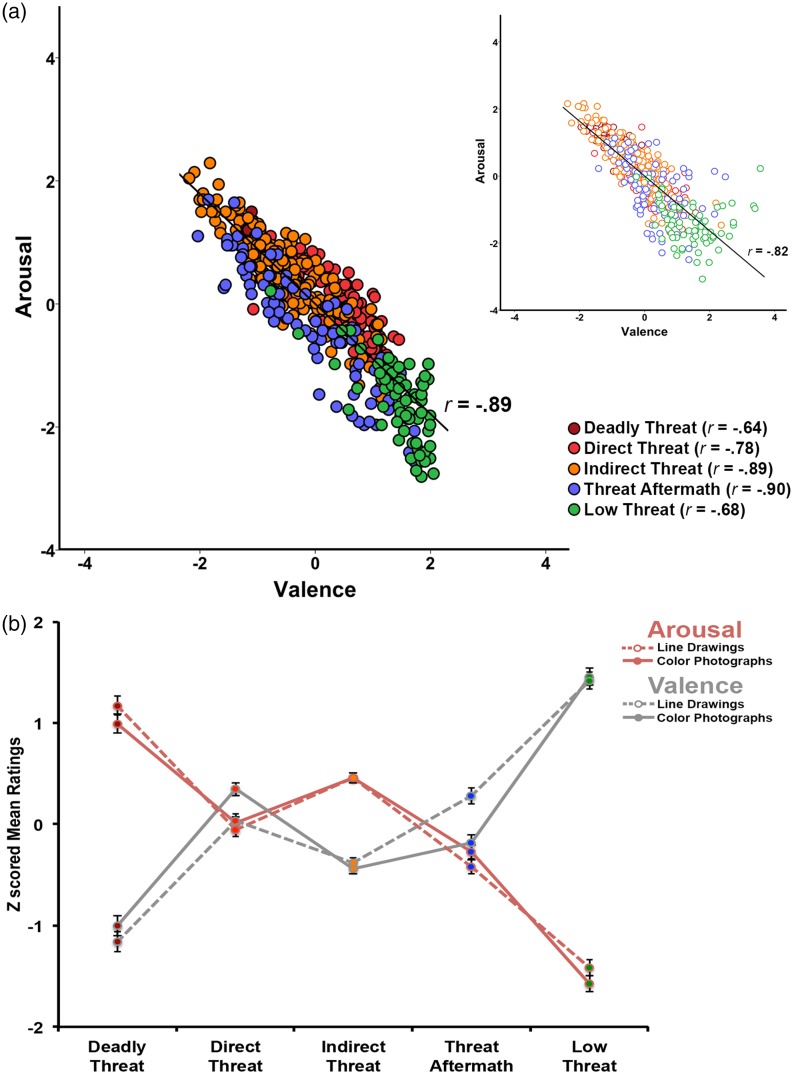
Study 2 validation results. (a) The relationship between valence and arousal ratings of color photographic scenes (left) with the relationship between valence and arousal ratings of line drawings of the matching scenes on the right for comparison. The colors correspond to image categories. (b) Means and *SE* of valence and arousal ratings for each image category for color photographic images and line-drawing images of matching scenes for comparison.

Because valence and arousal ratings for the color photographic images were collected on 6-point Likert scales as compared to 7-point Likert scales used in Study 2, all the ratings were *Z*-scored prior to analysis. Figure 7(a) shows the relationship between valence and arousal ratings of color photographic images. Similarly to line drawings, there was a strong negative linear relationship between valence and arousal ratings of color photographs, Pearson’s *r* = −.894, *p* < .001. To directly compare the scenes in color photographic and line drawing form on valence and arousal dimensions, we ran two 2(image type) × 5(image category) repeated measured ANOVAs using Type II calculation for obtaining sums of squares separately for each dimension (Figure 7(b)).

On arousal dimension, the analyses revealed no statistically significant main effect of image type (*F*(1,495) < .001, *p* = 1.000, *η^2^_p_* < .001), but a significant main effect of image category (*F*(4,495) = 179.78, *p* < .001, *η^2^_p_* = .592). All image categories were significantly different from each other as indicated by post hoc pairwise comparisons with a Bonferroni correction (all *p*s < .003). Deadly Threat images were rated as most arousing (*M* = 1.08, *SE* = .09), followed by Indirect Threat (*M* = .46, *SE* = .05), Direct Threat (*M* = −.02, *SE* = .06), Threat Aftermath (*M* = −.35, *SE* = .07), and Low Threat (*M* = −1.50, *SE* = .07). There was also a significant interaction between the image category and image type (*F*(4,495) = 3.56, *p* = .007, *η^2^_p_* = .028). Pairwise comparisons with a Bonferroni correction revealed that Deadly Threat and Low Threat images were rated as more arousing in line drawing compared to color photographic form (*t*(100) = 2.02, *p* = .044 and *t*(140) = 2.15, *p* = .032, respectively) while the opposite was observed for the Threat Aftermath images (*t*(162) = 2.00, *p* = .046). No statistically significant differences were found between line-drawing and color photographic images for Direct Threat and Indirect Threat images (both *p*s > .229, see Table 2 for descriptive statistics). It is worth noting that while the interaction effect was statistically significant (*p* = .007), the effect size observed was small (*η^2^_p_* = .028). Thus, the results reported here should be interpreted with caution.

**Table 2.** Descriptive Statistics for Valence and Arousal Ratings for Line Drawings and Color Photographic Images (data previously reported in Kveraga et al., 2015).

Line drawings							Color photographs				
		Arousal		Valence				Arousal		Valence	
		*M*	*SD*	*M*	*SD*			*M*	*SD*	*M*	*SD*
**Category**	Deadly Threat	1.17	.33	−1.16	.37	**Category**	Deadly Threat	.99	.25	−1.01	.24
	Direct Threat	−.06	.60	.04	.59		Direct Threat	.01	.51	.35	.63
	Indirect Threat	.46	.74	−.38	.79		Indirect Threat	.46	.72	−.44	.77
	Threat Aftermath	−.42	.88	.28	.82		Threat Aftermath	−.28	.98	−.12	.95
	Low Threat	−1.41	.64	1.42	.82		Low Threat	−1.58	.62	1.45	.52

On valence dimension, the analyses revealed no statistically significant main effect of image type (*F*(1,495) < .001, *p* = 1.000, *η^2^_p_* < .001), but a significant main effect of image category (*F*(4,495) = 145.40, *p* < .001, *η^2^_p_* = .540). All image categories were significantly different from each other (all *p*s < .001), except for no statistically significant difference between Direct Threat and Threat Aftermath images (*t*(199) = 1.55, *p* = .727) as indicated by post hoc pairwise comparisons with a Bonferroni correction. Deadly Threat images were rated as most negative (*M* = −1.08, *SE* = .09), followed by Indirect Threat (*M* = −.41, *SE* = .05), Direct Threat (*M* = .19, *SE* = .06), Threat Aftermath (*M* = .05, *SE* = .07), and Low Threat (*M* = 1.44, *SE* = .08). There was also a significant interaction between the image category and image type (*F*(4,495) = 21.34, *p* < .001, *η^2^_p_* = .147). Pairwise comparisons with a Bonferroni correction revealed that Direct Threat images were rated more negatively in line-drawing compared to color photographic form (*t*(236) = 5.69, *p* < .001). Similar but only marginally statistically significant effect was found for Deadly Threat images (*t*(100) = 1.85, *p* = .066). The opposite was observed for the Threat Aftermath images (*t*(140) = 6.94, *p* < .001). Specifically, Threat Aftermath images were rated less negatively in line-drawing compared to color photographic form. No statistically significant differences were found between line-drawing and color photographic images for Indirect Threat and Low Threat images (both *p*s > .214, see Table 2 for descriptive statistics).

## Discussion

In the present study, we demonstrated that human observers are able to finely discriminate different types of threat or merely negative situations from line-drawn scenes, even though line drawings lack many of the defining characteristics seen in the real world (such as color, most texture, most shading, and many background details). Specifically, we found that even though all of the images (with the exception of the Low Threat category) were negative, these scenes were keenly discriminated by the observers based on the perceived target of the threat (spatially directed towards the observer vs. towards someone else in the scene) as well as the temporal proximity of threat (happening right now or about to happen vs. something that happened sometime in the past). Previously, we reported that human observers are sensitive to different types of negative stimuli, with their evaluation of the stimuli differing sharply depending on the spatial and temporal direction of the threat using color photographic images (Kveraga et al., 2015). The present study replicated and extended our previous findings to line-drawn scenes.

The 500 open-access line-drawn images depicting scenes with different levels of threat created for the present study were rated by a sample of American young adults on three scales capturing spatial and temporal direction of the threat (how much harm there is to you, how much harm there is to someone else, and how much harm has already happened). Based on these ratings, 500 images were divided into five distinct categories: Deadly Threat (depicting humans with weapons pointing at the observer; *n* = 51), Direct Threat (depicting scenes posing high threat to the observer, *n* = 119), Indirect Threat (depicting scenes posing high threat to someone else; *n* = 117), Threat Aftermath (depicting scenes where harm—physical, psychological, or both—has already happened; *n* = 82), and Low Threat (depicting low level of threat either to the observer, or someone else; *n* = 71).

Immediate threats of personal harm should evoke a fight-or-flight response, while threats where the immediate danger has passed, or the threat is to others, might instead evoke an approach response, possibly to render assistance or to gather information that would enable observers to avoid a similar fate in the future (Kveraga, 2014; Oosterwijk, 2017). The ratings in the three groups (*Harm to you*, *Harm to other*, and *Past harm*) reflected the task, in that the image that received the highest ratings clearly depicted these spatial and temporal threat dimensions, with one exception. All three groups rated the Deadly Threat images (images depicting scenes of humans pointing deadly weapons—mostly guns—at the observer) highly, implying that they pose direct threat to the observer, as well as to someone else, and may have also caused past harm. In addition, Deadly Threat images evoked the lowest RT across all three groups. An alternative explanation of shorter RT could also indicate that the images belonging to the Deadly Threat category are easier to judge than the others, because they are less complex and also because it is easier to evaluate the situation from our point of view than from the point of view of someone else.

The valence and arousal dimensions had a strong negative linear relationship with each other both for the line-drawing and color photographic form of the matched scenes. Because all the images, with the exception of Low Threat category, were negative with a varying level and type of threat, this relationship is reasonable. The slightly stronger relationship for color photographic images as compared to line drawings of the matching scenes may be due to the fact that while the same participants rated both valence and arousal of the color photographic images, different participants rated valence and arousal of the line-drawing images. Line-drawing images depict the essence of the scene, while color photographic images provide more detail and a richer context.

It has been demonstrated that humans possess a natural ability to recognize and interpret line drawings and line drawings can evoke similar brain activity as color photographs (Walther et al., 2011). Our findings further demonstrate that young human observers show similar sensitivity for threat information in an image, which they can extract quickly whether they are presented with a color photographic image (Kveraga et al., 2015) or a simple line drawing. This indicates that the gist of the scene is extracted and used in categorizing threat and merely negative stimuli, in color photographs and line drawings alike, as the latter are missing much detail, color and background information available in the former. Our findings make sense in light of evolutionary pressures, as the human (and its mammalian predecessors’) visual system had to be able to extract the form of threatening stimuli in widely varying visibility conditions quickly and automatically to promote survival. Among such stimuli, snakes, spiders, and angry faces are thought to be especially important cues of potential danger (Öhman et al., 2001; Öhman & Mineka, 2001), with snakes being the primary predator of humans’ primate predecessors (Isbell, 2006; Le et al., 2016). However, our results revealed no natural predators in the Deadly Threat category, which was dominated by images of humans pointing weapons at the observer. While we did not explicitly set out to test whether these stimuli would be recognized faster than natural predators, or unarmed, angry humans, the ratings and the RTs clearly suggest that humans pointing weapons, including guns, are considered significantly more dangerous than natural predators. This may stem from most observers’ surroundings, upbringing and limited experience with natural threats. While they may not have had any personal experience with people pointing guns at them, societal exposure to such stimuli via the media may have been sufficient to encode such evolutionarily modern stimuli as more dangerous than natural predators.

### Potential Uses of Threat-Depicting and Negative Line Drawings in Research

Compared to photographic images, line-drawing images are much more easily manipulated without disrupting the natural image statistics, making them a useful tool for studying objects and scenes (Biederman & Ju, 1988; Biederman et al., 1982; Kveraga et al., 2007; Palmer, 1975; Snodgrass & Vanderwart, 1980; Walther et al., 2011). In addition, unlike color photographic scenes, line-drawn scenes offer better control over image statistics across image categories. For example, a color photographic scene depicting a man with a gun pointed at the observer (Deadly Threat) differs from a scene depicting a gravely injured person (Threat Aftermath) not only in the threat context but also in presence (or absence) of color and textural visual cues, such as blood. On the other hand, line-drawn images provide only the gist of the scene across different image categories and thus offer better experimental control. This has been shown to be important in, for example, facial expression research, where anger expressions are detected more quickly when the facial stimuli are identical in their low-level components (e.g., schematic, line-drawn faces), but the evidence is mixed when face photographs are used (Öhman et al., 2012). Thus, our line-drawing image set could be used to study threat and emotion perception, either in original or altered form, for example, through visual pathway biasing (Kveraga et al., 2007; Thomas, Kveraga, Huberle, Karnath, & Bar, 2012), fragmenting (Biederman & Ju, 1988), grouping into global/local ensembles (Walther et al., 2011), manipulating object-context (Palmer, 1975), object-agent (Correll, Park, Judd, & Wittenbrink, 2002), object-location (Biederman et al., 1982) congruency, or facial expressions (Öhman et al., 2012).

Furthermore, given that our line-drawing image set contains distinct image categories with varying threat loadings, differentiating negative, threatening images (Deadly Threat, Direct Threat, and Indirect Threat) from merely negative images (Threat Aftermath), it may also be useful for studying clinical populations showing abnormal sensitivity to threat stimuli. For example, various studies demonstrated that anxious individuals exhibit selective attention for threat-related stimuli (Eysenck, Derakshan, Santos, & Calvo, 2007; Mogg & Bradley, 1999; Mogg, Millar, & Bradley, 2000), spend more time on such stimuli (Fox, Russo, Bowles, & Dutton, 2001), and have difficulty in shifting their attention away from threat (Klumpp & Amir, 2009; Yiend & Mathews, 2001). In a recent fMRI study (Im et al., 2017), we utilized visual pathway biasing manipulation (Kveraga et al., 2007; Thomas et al., 2012) and found that trait anxiety differentially affected perception of clear and ambiguous threat cues from faces presented to the magnocellular or parvocellular visual pathways and correlated with amygdala activity in lateralized fashion. The reported abnormalities in threat processing by anxious individuals can be studied further by examining their responses to the different types of negative and threatening images (Deadly Threat, Direct Threat and Indirect Threat) compared to responses to merely negative images (Threat Aftermath).

### Limitations and Future Directions

Among the limitations of the study is the use of young adult U.S. student samples. Future work should investigate larger scale samples of young, as well as middle-aged and older adults in order to increase generalizability of the findings reported here. In addition, there is an unbalanced number of images per category in the image set described here. However, the set offers a fairly large pool of images to choose from, and a balanced design among the categories can be accomplished by selecting image subsets. Researchers will be able to select a sample of stimuli corresponding to their design needs from the entire image set, either by using the ratings reported here or by rerating the sample in their own population, with the same or new rating parameters relevant to their research question. Furthermore, even though a reasonably stable relationship has been observed between psychophysiological responses such as heart rate, skin conductance, facial EMG, and the startle reflex as compared to subjective affective ratings (Lang, Bradley, & Cuthbert, 1990), it is nevertheless necessary to acquire affective responses to our line-drawing images that go beyond self-reported subjective ratings using psychophysiological reactions (Cacioppo, Berntson, Larsen, Poehlmann, & Ito, 2000), fMRI (Kveraga et al., 2015; Moriguchi et al., 2011), and magnetoencephalography (MEG: Panichello, Kveraga, Chaumon, Bar, & Barrett, 2017). Finally, using IAPS images, cross-cultural studies conducted around the world (Deak, Csenki, & Revesz, 2010; Drace, Efendic, Kusturica, & Landzo, 2013; Dufey, Fernandez, & Mayol, 2011; Lasaitis, Ribeiro, & Bueno, 2008; Lohani, Gupta, & Srinivasan, 2013) revealed major similarities as well as subtle cultural differences in terms of valence and arousal ratings. Thus, future studies will have to evaluate the cross-cultural validity of the current set of images.

Future work on the line-drawing image set presented here should investigate the following: (a) larger scale samples of young, middle-aged, and older adults that might reveal age-related differences in processing affectively relevant images in the set; (b) the validity and reliability of the set across clinical populations, such as populations suffering from anxiety disorders; (c) the validity and reliability of the set across cultures; and (d) affective responses to the set that go beyond self-reported subjective ratings, such as heart rate, skin conductance, and facial EMG.

## Conclusions

We have previously demonstrated that young human observers are keenly sensitive to the spatial and temporal directions of threat in color photographic visual scenes (Kveraga et al., 2015). Here, we extended our previous work by demonstrating that young human observers are just as sensitive to spatial and temporal directions of threat, even when it is presented as hand-traced line drawings of the same visual scenes. This suggests that, even though line drawings are much more visually impoverished as compared to color photographic images, they nevertheless appear to capture essential features that allow human observers to differentiate the scenes according to their threat value. Because a line-drawing format of an image allows for easier manipulation of the image content, our line-drawing stimulus set offers a multitude of possibilities for future research on threat perception. A more nuanced understanding of threat perception is not only of considerable theoretical interest, but has practical implications for the safety and well-being of healthy and clinical populations.

## Supplemental Material

Supplementary Material table and figures

## Author Note

This work was presented at the seventeenth annual meeting of Vision Sciences Society (VSS 2017) held in St. Pete Beach, FL, in May 2017. Institutional Review Board exemption was obtained before the beginning of the study.

## Notes

1 The interrater reliability for each rating group before removing data of eight participants (*n* = 20 each) was .972, .969, and .950 respectively.

2 If an image fitted more than one pattern, the pattern producing a more significant fit was selected.

3 For arousal ratings, one participant’s responses were significantly different from the responses of other participants. Thus, data of this participant were removed from further analysis. Interreliability for arousal ratings before removing these data was .956.

## References

[bibr1-2041669518755806] Bannerman R. L. Milders M. de Gelder B. Sahraie A. (2009) Orienting to threat: Faster localization of fearful facial expressions and body postures revealed by saccadic eye movements. Proceedings of the Royal Society of Sciences: B Biological Sciences 276: 1635–1641. doi: http://dx.doi.org/10.1098/rspb.2008.1744. 10.1098/rspb.2008.1744 PMC2660988 19203922

[bibr2-2041669518755806] Barrett L. F. (2006) Valence as a basic building block of emotional life. Journal of Research in Personality 40: 35–55. doi: http://dx.doi.org/10.1016/j.jrp.2005.08.006.

[bibr3-2041669518755806] Barrett L. F. Bliss-Moreau E. (2009) Affect as a psychological primitive. Advances in Experimental Social Psychology 41: 167–218. doi: https://doi.org/10.1016/S0065-2601(08)00404-8. 20552040 10.1016/S0065-2601(08)00404-8 PMC2884406

[bibr4-2041669518755806] Barrett L. F. Russell J. A. (1999) Structure of current affect. Current Directions in Psychological Science 8: 10–14. doi: https://doi.org/10.1111/1467-8721.00003.

[bibr5-2041669518755806] Becker D. V. Anderson U. S. Mortensen C. R. Neufeld S. L. Neel R. (2011) The face in the crowd effect unconfounded: Happy faces, not angry faces, are more efficiently detected in single- and multiple-target visual search tasks. Journal of Experimental Psychology: General 140: 637–659. doi: https://doi.org/10.1037/a0024060. 21744984 10.1037/a0024060

[bibr6-2041669518755806] Becker D. V. Srinivasan N. (2014) The vividness of the happy face. Current Directions in Psychological Science 23: 189–194. doi: https://doi.org/10.1177/0963721414533702.

[bibr7-2041669518755806] Biederman I. Ju G. (1988) Surface versus edge-based determinants of visual recognition. Cognitive Psychology 14: 143–177. doi: https://doi.org/10.1016/0010-0285(82)90007-X. 10.1016/0010-0285(88)90024-2 3338267

[bibr8-2041669518755806] Biederman I. Mezzanotte R. J. Rabinowitz J. C. (1982) Scene perception: Detecting and judging objects undergoing relational violations. Cognitive Psychology 14: 143–177. doi: https://doi.org/10.1016/0010-0285(82)90007-X. 7083801 10.1016/0010-0285(82)90007-x

[bibr9-2041669518755806] Blanchette I. (2006) Snakes, spiders, guns, and syringes: How specific are evolutionary constraints on the detection of threatening stimuli? The Quarterly Journal of Experimental Psychology 59: 1484–1504. doi: https://doi.org/10.1080/02724980543000204. 16846972 10.1080/02724980543000204

[bibr10-2041669518755806] Brosch T. Pourtois G. Sander D. (2010) The perception and categorisation of emotional stimuli: A review. Cognition and Emotion 24: 377–400. doi: https://doi.org/10.1080/02699930902975754.

[bibr11-2041669518755806] Cacioppo J. T. Berntson G. G. Larsen J. T. Poehlmann K. M. Ito T. A. (2000) The psychophysiology of emotion. In: Lewis M. Haviland-Jones J. M. (eds) Handbook of emotions, 2nd ed New York, NY: Guilford Press, pp. 173–191.

[bibr12-2041669518755806] Correll J. Park B. Judd C. M. Wittenbrink B. (2002) The police officer's dilemma: Using ethnicity to disambiguate potentially threatening individuals. Journal of Personality and Social Psychology 83: 1314–1329. doi: https://doi.org/10.1037/0022-3514.83.6.1314. 12500813

[bibr13-2041669518755806] Dan-Glauser E. S. Scherer K. R. (2011) The Geneva affective picture database (GAPED): A new 730-picture database focusing on valence and normative significance. Behavioral Research 43: 468–477. 10.3758/s13428-011-0064-1 21431997

[bibr14-2041669518755806] Deak A. Csenki L. Revesz G. (2010) Hungarian ratings for the International Affective Picture System (IAPS): A cross-cultural comparison. Empirical Text and Culture Research 4: 90–101.

[bibr15-2041669518755806] Drace S. Efendic E. Kusturica M. Landzo L. (2013) Cross-cultural validation of the “International Affective Picture System” (IAPS) on a sample from Bosnia and Herzegovina. Psihologija 46: 17–26.

[bibr16-2041669518755806] Duckworth K. L. Bargh J. A. Garcia M. Chaiken S. (2002) The automatic evaluation of novel stimuli. Psychological Science 13: 513–519. doi: https://doi.org/10.1111/1467-9280.00490. 12430834 10.1111/1467-9280.00490

[bibr17-2041669518755806] Dufey M. Fernandez A. M. Mayol R. (2011) Adding support to cross-cultural emotional assessment: Validation of the International Affective Picture System in a Chilean sample. Universitas Psychologica 10: 521–533.

[bibr18-2041669518755806] Eastwood J. D. Smilek D. Merikle P. M. (2001) Differential attentional guidance by unattended faces expressing positive and negative emotion. Perception and Psychophysics 63: 1004–1013. doi: https://doi.org/10.3758/BF03194519. 11578045 10.3758/bf03194519

[bibr19-2041669518755806] Eysenck M. W. Derakshan N. Santos R. Calvo M. G. (2007) Anxiety and cognitive performance: Attentional control theory. Emotion 7: 336–353. doi: https://doi.org/10.1037/1528-3542.7.2.336. 17516812 10.1037/1528-3542.7.2.336

[bibr20-2041669518755806] Fox E. Russo R. Bowles R. J. Dutton K. (2001) Do threatening stimuli draw or hold visual attentional in subclinical anxiety? Journal of Experimental Psychology: General 130: 681–700. doi: https://doi.org/10.1037/0096-3445.130.4.681. 11757875 PMC1924776

[bibr21-2041669518755806] Hansen C. H. Hansen R. D. (1988) Finding the face in the crowd – An anger superiority effect. Journal of Personality and Social Pscyhology 54: 917–924. doi: https://doi.org/10.1037/0022-3514.54.6.917. 10.1037//0022-3514.54.6.917 3397866

[bibr22-2041669518755806] Horstmann G. (2007) Preattentive face processing: What do visual search experiments with schematic faces tell us? Visual Cognition 15: 799–833. doi: https://doi.org/10.1080/13506280600892798.

[bibr23-2041669518755806] Im, H. Y., Adams R. B. Jr., Boshyan, J., Ward, N., Cushing, C. A., & Kveraga, K. (2017). Observer's anxiety facilitates magnocellular processing of clear facial threat cues, but impairs parvocellular processing of ambiguous facial threat cues. *Scientific Reports*, *7*, 15151. doi: 10.1038/s41598-017-15495-2. 10.1038/s41598-017-15495-2 PMC5680327 29123215

[bibr24-2041669518755806] Isbell L. A. (2006) Snakes as agents of evolutionary change in primate brains. Journal of Human Evolution 51: 1–35. doi: https://doi.org/10.1016/j.jhevol.2005.12.012. 16545427 10.1016/j.jhevol.2005.12.012

[bibr25-2041669518755806] Klumpp H. Amir N. (2009) Examination of vigilance and disengagement of threat in social anxiety with a probe detection task. Anxiety, Stress, and Coping 22: 283–296. doi: https://doi.org/10.1080/10615800802449602. 10.1080/10615800802449602 PMC3712328 19253172

[bibr26-2041669518755806] Kurdi B. Lozano S. Banaji M. R. (2017) Introducing the Open Affective Standardized Image Set (OASIS). Behavior Research Methods 49: 457–470. doi: http://dx.doi.org/10.3758/s13428-016-0715-3. 26907748 10.3758/s13428-016-0715-3

[bibr27-2041669518755806] Kveraga K. (2014) Threat perception in visual scenes: Dimensions, action and neural dynamics. In: Kveraga K. Bar M. (eds) Scene Vision: Making sense of what we see, Cambridge, MA: MIT Press, pp. 291–307.

[bibr28-2041669518755806] Kveraga K. Boshyan J. Adams R. B. Mote J. Betz N. Ward N. Barrett L. F. (2015) If it bleeds, it leads: Separating threat from mere negativity. Social Cognitive Affective Neuroscience 10: 28–35. doi: https://doi.org/10.1093/scan/nsu007. 24493851 10.1093/scan/nsu007 PMC4994838

[bibr29-2041669518755806] Kveraga K. Boshyan J. Bar M. (2007) Magnocellular projections as the trigger of top-down facilitation in recognition. Journal of Neuroscience 27: 13232–13240. doi: https://doi.org/10.1523/JNEUROSCI.3481-07.2007. 18045917 10.1523/JNEUROSCI.3481-07.2007 PMC6673387

[bibr30-2041669518755806] Lang P. J. Bradley M. M. Cuthbert B. N. (1990) Emotion, attention, and the startle reflex. Psychological Review 97: 377–395. doi: https://doi.org/10.1037/0033-295X.97.3.377. 2200076

[bibr31-2041669518755806] Lang, P. J., Bradley, M. M, & Cuthbert, B. N. (2005). *International affective picture system (IAPS): Affective ratings of pictures and instruction manual.* Technical Report No A-6. University of Florida. Gainesville, FL.

[bibr32-2041669518755806] Larsen, R. J., & Diener, E. (1992). Promises and problems with the circumplex model of emotion. In M. S. Clark (Ed.), *Review of personality and social psychology: Emotion* (Vol. 13, pp. 25–59). Newbury Park, CA: Sage.

[bibr33-2041669518755806] Lasaitis C. Ribeiro R. L. Bueno O. F. A. (2008) Brazilian norms for the International Affective Picture System (IAPS): Comparison of the affective ratings for new stimuli between Brazilian and North-American subjects. Jornal Brasileiro De Psiquiatria 57: 270–275.

[bibr34-2041669518755806] Le Q. V. Isbell L. A. Matsumoto J. Le V. Q. Nishimaru H. Hori E. Nishijo H. (2016) Snakes elicit earlier, and monkey faces, later, gamma oscillations in macaque pulvinar neurons. Scientific Reports 6: 20595–20604. doi: https://doi.org/10.1038/srep20595. 26854087 10.1038/srep20595 PMC4744932

[bibr35-2041669518755806] Le Q. V. Isbell L. A. Matsumoto J. Nguyen M. Hori E. Maior R. S. Nishijo H. (2013) Pulvinar neurons reveal neurobiological evidence of past selection for rapid detection of snakes. Proceedings of the National Academy of Sciences of the United States of America 110: 19000–19005. doi: https://doi.org/10.1073/pnas.1312648110. 24167268 10.1073/pnas.1312648110 PMC3839741

[bibr36-2041669518755806] LeDoux J. (2012) Rethinking the emotional brain. Neuron 73: 653–676. doi: https://doi.org/10.1016/j.neuron.2012.02.004. 22365542 10.1016/j.neuron.2012.02.004 PMC3625946

[bibr37-2041669518755806] LoBue V. (2009) More than just a face in the crowd: Detection of emotional facial expressions in young children and adults. Developmental Science 12: 305–313. doi: https://doi.org/10.1111/j.1467-7687.2008.00767.x. 19143803 10.1111/j.1467-7687.2008.00767.x

[bibr38-2041669518755806] LoBue V. (2010a) And along came a spider: Superior detection of spiders in children and adults. Journal of Experimental Child Psychology 107: 59–66. doi: https://doi.org/10.1016/j.jecp.2010.04.005. 20529694 10.1016/j.jecp.2010.04.005

[bibr39-2041669518755806] LoBue V. (2010b) What's so scary about needles and knives? Examining the role of experience in threat detection. Cognition and Emotion 24: 80–87. doi: https://doi.org/10.1080/02699930802542308.

[bibr40-2041669518755806] LoBue V. DeLoache J. S. (2008) Detectiong the snake in the grass: Attention to ffear-relevant stimuli by adults and young children. Psychological Science 19: 284–289. doi: https://doi.org/10.1111/j.1467-9280.2008.02081.x. 18315802 10.1111/j.1467-9280.2008.02081.x

[bibr41-2041669518755806] LoBue V. DeLoache J. S. (2010) Superior detection of threat-relevant stimuli in infancy. Developmental Science 13: 221–228. doi: https://doi.org/10.1111/j.1467-7687.2009.00872.x. 20121878 10.1111/j.1467-7687.2009.00872.x

[bibr42-2041669518755806] LoBue V. Rakison D. H. DeLoache J. S. (2010) Threat perception across the life span: Evidence for multiple converging pathways. Current Directions in Psychological Science 19: 375–379. doi: https://doi.org/10.1177/0963721410388801.

[bibr43-2041669518755806] Lohani M. Gupta R. Srinivasan N. (2013) Cross-culturalevaluation of the International Affective Picture System on an Indian sample. Psychological Studies 58: 233–241.

[bibr44-2041669518755806] Mogg K. Bradley B. P. (1999) Orienting of attention to threatening facial expressions presented under conditions of restricted awarness. Cognition and Emotion 13: 713–740. doi: https://doi.org/10.1080/026999399379050.

[bibr45-2041669518755806] Mogg K. Millar N. Bradley B. P. (2000) Biases in eye movements to threatening facial expressions in feneralized anxiety disorder and depressive disorder. Journal of Abnormal Psychology 109: 695–704. doi: https://doi.org/10.1037/0021-843X.109.4.695. 11195993 10.1037//0021-843x.109.4.695

[bibr46-2041669518755806] Moriguchi Y. Negreira A. Weierich M. Dautoff R. Dickerson B. C. Wright C. I. Barrett L. F. (2011) Differential hemodynamic response in affective circuitry with aging: An fMRI study of novelty, valence, and arousal. Journal of Cognitive Neuroscience 23: 1027–1041. doi: https://doi.org/10.1162/jocn.2010.21527. 20521849 10.1162/jocn.2010.21527 PMC3141584

[bibr47-2041669518755806] New J. Cosmides L. Tooby J. (2007) Category-specific attention for animals reflects ancestral priorities, not expertise. Proceedings of the National Academy of Sciences of the United States of America 104: 16598–16603. doi: https://doi.org/10.1073/pnas.0703913104. 17909181 10.1073/pnas.0703913104 PMC2034212

[bibr48-2041669518755806] Nummenmaa L. Hyona J. Calvo M. G. (2009) Emotional scene content drives the saccade generation system reflecively. Journal of Experimental Psychology: Human Perception and Performance 35: 305–323. doi: https://doi.org/10.1037/a0013626. 19331490 10.1037/a0013626

[bibr49-2041669518755806] Öhman A. Flykt A. Esteves F. (2001) Emotion drives attention: Detecting the snake in the grass. Journal of Experimental Psychology: General 130: 466–478. doi: https://doi.org/10.1037/0096-3445.130.3.466. 11561921 10.1037/0096-3445.130.3.466

[bibr50-2041669518755806] Öhman A. Mineka S. (2001) Fears, phobias, and preparedness: Toward an evolved module of fear and fear learning. Psychological Review 108: 483–522. doi: https://doi.org/10.1037/0033-295X.108.3.483. 11488376 10.1037/0033-295x.108.3.483

[bibr51-2041669518755806] Öhman A. Soares S. C. Juth P. Lindström B. R. Esteves F. (2012) Evolutionary derived modulations of attention to two common fear stimuli: Serpents and hostile humans. Journal of Cognitive Psychology 24: 17–32. doi: https://doi.org/10.1080/20445911.2011.629603.

[bibr52-2041669518755806] Oosterwijk S. (2017) Choosing the negative: A behavioral demonstration of morbid curiosity. PLoS One 12: e0178399 doi: https://doi.org/10.1371/journal.pone.0178399. 28683147 10.1371/journal.pone.0178399 PMC5500011

[bibr53-2041669518755806] Palmer S. E. (1975) The effectsof contextual scenes on the identification of objects. Memory & Cognition 3: 519–526. doi: https://doi.org/10.3758/BF03197524. 24203874 10.3758/BF03197524

[bibr54-2041669518755806] Panichello M. T. Kveraga K. Chaumon M. Bar M. Barrett L. F. (2017) Internal valence modulates the speed of object recognition. Scientific Reports. Advance online publication. doi: https://doi.org/10.1038/s41598-017-00385-4. 10.1038/s41598-017-00385-4 PMC5428282 28336933

[bibr55-2041669518755806] Ratcliff R. (1993) Methods for dealing with reaction time outliers. Psychological Bulletin 114: 510–532. doi: https://doi.org/10.1037/0033-2909.114.3.510. 8272468 10.1037/0033-2909.114.3.510

[bibr56-2041669518755806] Russell J. A. (1980) A circumplex model of affect. Journal of Personality and Social Pscyhology 39: 1161–1178. doi: https://doi.org/10.1037/h0077714.

[bibr57-2041669518755806] Schupp H. T. Öhman A. Junghofer M. Weike A. I. Stockburger J. Hamm A. O. (2004) The facilitated processing of threatening faces: An ERP analysis. Emotion 4: 189–200. doi: https://doi.org/10.1037/1528-3542.4.2.189. 15222855 10.1037/1528-3542.4.2.189

[bibr58-2041669518755806] Seligman M. E. (1971) Phobias and preparedness. Behavior Therapy 2: 307–320. doi: https://doi.org/10.1016/S0005-7894(71)80064-3. 10.1016/j.beth.2016.08.006 27816071

[bibr59-2041669518755806] Snodgrass J. G. Vanderwart M. (1980) A standardized set of 260 pictures: Norms for name agreement, image agreement, familiarity, and visual complexity. Journal of Experimental Psychology: Human Learning and Memory 6: 174–215. doi: https://doi.org/10.1037/0278-7393.6.2.174. 7373248 10.1037//0278-7393.6.2.174

[bibr60-2041669518755806] Thomas C. Kveraga K. Huberle E. Karnath H.-O. Bar M. (2012) Enabling global processing in simultanagnosia by psychophysical biasing of visual pathways. Brain 135: 1578–1585. doi: https://doi.org/10.1093/brain/aws066. 22418740 10.1093/brain/aws066 PMC3338926

[bibr61-2041669518755806] Tipples J. Atkinson A. P. Young A. W. (2002) The eyebrow frown: A salient social signal. Emotion 2: 288–296. doi: https://doi.org/10.1037/1528-3542.2.3.288. 12899361 10.1037/1528-3542.2.3.288

[bibr62-2041669518755806] Walther D. B. Chai B. Caddigan E. Beck D. M. Li F.-F. (2011) Simple line drawings suffice for functional MRI decoding of natural scene categories. Proceedings of the National Academy of Sciences of the United States of America 108: 9661–9666. doi: https://doi.org/10.1073/pnas.1015666108. 21593417 10.1073/pnas.1015666108 PMC3111263

[bibr63-2041669518755806] Watson D. Tellegen A. (1985) Toward a consensual structure of mood. Psychological Bulletin 98: 219–223. doi: https://doi.org/10.1037/0033-2909.98.2.219. 3901060 10.1037//0033-2909.98.2.219

[bibr64-2041669518755806] Yiend J. Mathews A. (2001) Anxiety and attention to threatening pictures. Quarterly Journal of Experimental Psychology: A Human Experimental Psychology 54: 665–681. doi: https://doi.org/10.1080/713755991. 11548029 10.1080/713755991

