# Supplementary material for: Line-Drawn Scenes Provide Sufficient Information for Discrimination of Threat and Mere Negativity
Source: Iperception. 2018 Feb 12;9(1):2041669518755806. doi: 10.1177/2041669518755806 (PMC5950935; doi:10.1177/2041669518755806)

Supplemental Table 1. Demographic information broken down by the rating group for total number of participants (a) and participants whose data were used in the current study (b).

	a) All (n = 60)			b) Analyzed (n = 52)		
	**Group 1**	**Group 2**	**Group 3**	**Group 1**	**Group 2**	**Group 3**
**N**	20	20	20	18	17	17
**Age**						
Mean	20.40	19.35	19.00	20.11	19.47	18.88
Median	20	19	19	20	19	19
Std. Deviation	2.64	1.42	1.17	2.00	1.50	0.99
**Sex**						
Male	11	9	8	11	8	8
Female	9	11	12	7	9	9
**Race**						
White	13	12	10	12	10	9
Afr. American	0	0	1	0	0	1
Asian	6	5	7	5	5	5
Other	1	3	2	1	2	2

Supplemental Table 2. Inter-rater reliability for each rating group before and after the exclusion of data of eight participants.

Group	Participated	Excluded	Inter-rater Reliability Before	After
1	20	2	.972 (n=20)	.986 (n=18)
2	20	3	.969 (n=20)	.983 (n=17)
3	20	3	.950 (n=20)	.969 (n=17)
Total	60	8		Mean α = .979

In Group 1, participant-group correlations for two participants were negative (r = -.286 and r = -.054; the rest of the r's above .816). Removing these two participants from Group 1 increased inter-rater reliability from .972 to .986. In Group 2, three participants with poor correlations were identified (r = .255, r = .280 and r = .321; the rest of the r's above .702). Removing these three participants from Group 2 increased inter-rater reliability from r = .969 to r = .983. Similarly, in Group 3, another three participants were identified (r = .149, r = .259, r = .273; the rest of the r's above .700). After removing these 3 participants, the inter-rater reliability of Group 3 also increased from .950 to .969. It is important to note that two participants with significantly faster responses also had poor participant-group correlations, offering further confirmation of our decision to exclude their data from further analysis.

After removing data of the eight participants described above, the number of ratings for each image provided by participants in Group 1 ranged from 13 to 18, with a mean of 17.33 ratings (SD = .88) per image; in Group 2 it ranged from 11 to 17, with a mean of 16.32 ratings (SD = .97) per image; and in Group 3 it ranged from 12 to 17, with a mean of 16.41 ratings (SD = .89) per image.

Face validity

In order to evaluate the face validity of ratings provided by the three groups we further investigated images with the highest and lowest ratings and those with the largest and smallest standard deviation, indicating low and high levels of agreement, respectively (Supplemental Figure 1a). The highest rating from Group 1 (harm to you) was obtained for image D086 (M = 5.83, SD = .38), which depicts two masked men pointing guns at the observer, and the lowest rating was obtained for image N005 (M = 1.06, SD = .24), which depicts a smiling soldier hugging a woman. This image also had the lowest standard deviation, showing the highest agreement among the raters in Group 1. For Group 2 (harm to other), the highest rating was obtained for image I062 (M = 5.82, SD = .39), which depicts a man pointing a gun to his own head, and the lowest rating was obtained for image N018 (M = 1.00, SD = .00), depicting a woman walking a dog on a leash. This image had a standard deviation of 0, showing the highest agreement among the raters in Group 2. For Group 3 (past harm), the highest rating was obtained for image I031 (M = 5.71, SD = .59), depicting an alligator with a severed human forearm in its jaws, and the lowest rating was obtained for image N007 (M = 1.06, SD = .25), which depicts three men having a drink together. This image also had the highest agreement among the raters in Group 3, as indicated by the lowest standard deviation. Image D023, which depicts an insect (mantis) in a threat position facing the observer, had the highest standard deviation (1.59, M = 1.59) indicating lowest agreement among the raters in Group 1. In Group 2, image D076, depicting an eel displaying its teeth and facing the observer, had the highest standard deviation (1.44, M = 4.29). Finally, in Group 3 image NG046 had the highest standard deviation (1.44, M = 4.07), depicting a dead alligator with a tool sticking out of its back.

In addition, we plotted mean ratings of each group by percent of responses grouped by five image categories (Deadly Threat, Direct Threat, Indirect Threat, Threat Aftermath, and Low Threat). Percent of responses was calculated separately for each image category by dividing frequency of mean ratings by the number of stimuli in each image category. Supplemental Figure 1b shows that, as expected, participants in Group 1 rated Direct Threat and Deadly Threat images the highest, participants in Group 2 rated Indirect Threat and Deadly Threat images the highest, participants in Group 3 rated Threat Aftermath images the highest, while all three groups of participants rated the Low Threat images the lowest.

Overall, these analyses suggest that each group was keenly sensitive to their respective task and able to extract the relevant information from the line drawings.

Univariate distributions

The distribution of the image-wise means and standard deviations is shown in Supplemental Figure 2 (panels a and b respectively). The ratings provided by all three groups showed good usage of the entire range of the scale (ratings ranged from 1.06 to 5.83 for Group 1; from 1 to 5.88 for Group 2; and from 1.06 to 5.71 for Group 3). The overall mean rating for Group 1 was 3.25, and the overall median standard deviation was .80. Overall, the distribution of the mean ratings from Group 1 was slightly bimodal with Low Threat and Threat Aftermath images rated lower as compared to Direct Threat and Deadly Threat images, which were rated higher. Low Threat images also showed highest agreement as compared to other image categories. The overall mean rating for Group 2 was 3.65, and the median standard deviation was .73. The distribution of Group 2 ratings looked somewhat negatively skewed, with Low Threat and Threat Aftermath images receiving lower ratings as compared to higher ratings for Indirect Threat and Deadly Threat images. Overall, participants in Group 2 showed higher agreement as

compared to Group 1. Finally, the mean rating for Group 3 was 3.43, with the median standard deviation of .85, with fairly normal distribution with slight negative skew. Once again, Low Threat images had the lowest ratings and Threat Aftermath images received the highest ratings. Participants in Group 3 also showed the highest agreement for Low Threat images.

Relationship between means and standard deviations

We also investigated the relationship between image-wise means and image-wise standard deviations for each of the three rating groups by fitting linear, quadratic, and cubic regressions to the data with image-wise means as predictors and image-wise standard deviations as criterion variable (Supplemental Figure 2c).

For Group 1 the scatterplot (Supplemental Figure 2c, left panel) displays an inverted U-shaped relationship between means and standard deviations, indicating that standard deviations were lowest at the low end of the scale (Low Threat images), became higher as the mean increased (Indirect Threat and Threat Aftermath images), and leveled off again at the very high end of the scale (Direct Threat and Deadly Threat images). Given that the task for Group 1 was to rate each image based on how much harm to them (to the observer) it depicted, this kind of relationship is reasonable: both Low Threat and Deadly/Direct Threat images depicted relatively clear content (either low or high potential harm to the observer respectively), while images depicting Indirect Threat and Threat Aftermath left more room for interpretation, leading to lower agreement, and thus higher standard deviation. The visual impression of an inverted U-shaped relationship was further confirmed by the fact that a quadratic regression provided the best fit to the data, with a fairly strong relationship between means and standard deviations, $R^2 = .365$.

Group 2 scatterplot displays a similar picture, although the inverted U-shaped relationship between means and standard deviations is flatter (Supplemental Figure 2c, middle panel). Once again, quadratic regression provided the best fit to the data, with a relatively lower relationship between the means and standard deviations, R^2 = .177. However, the scatterplot reveals that unlike Group 1, Group 2 participants showed more uniform agreement for the images across all categories, with the exception of the images that fall at both extremes of the scale. Participants showed an especially high level of agreement for these extreme images (i.e., Low Threat images at the low end of the scale and Indirect Threat and Deadly Threat images at the high end of the scale). This can explain why a weaker quadratic trend might have arisen.

In contrast, the scatterplot for Group 3 displays a somewhat positive linear relationship. For Group 3, Low Threat images were rated the lowest with highest agreement (lowest standard deviations) and the Threat Aftermath and Indirect Threat images rated the highest with least agreement (highest standard deviations) among the raters (Supplemental Figure 2c, right panel). This impression was further confirmed by the fact that linear regression provided the best fit to the data, with a relatively weak relationship between the means and standard deviations, R^2 = .172. While the Low Threat images were easiest to assess in terms of the probability of past harm having already occurred (since no harm was depicted in these images), the rest of the images presented more room for interpretation, thus leading to less agreement.

Supplemental Figure 1. Face validity of ratings provided by the three groups. (a) Images that received the highest and lowest mean ratings and had the highest and lowest standard deviation, indicating low and high levels of agreement respectively for each rating group. (b) Mean ratings of each group by percent of responses grouped by five image categories (Deadly Threat, Direct Threat, Indirect Threat, Threat Aftermath, and Low Threat). Percent of responses was calculated separately for each image category by dividing frequency of mean ratings by the number of stimuli in each image category. The figure demonstrates that each group was keenly sensitive to their respective task and able to extract the relevant information from the line drawings.

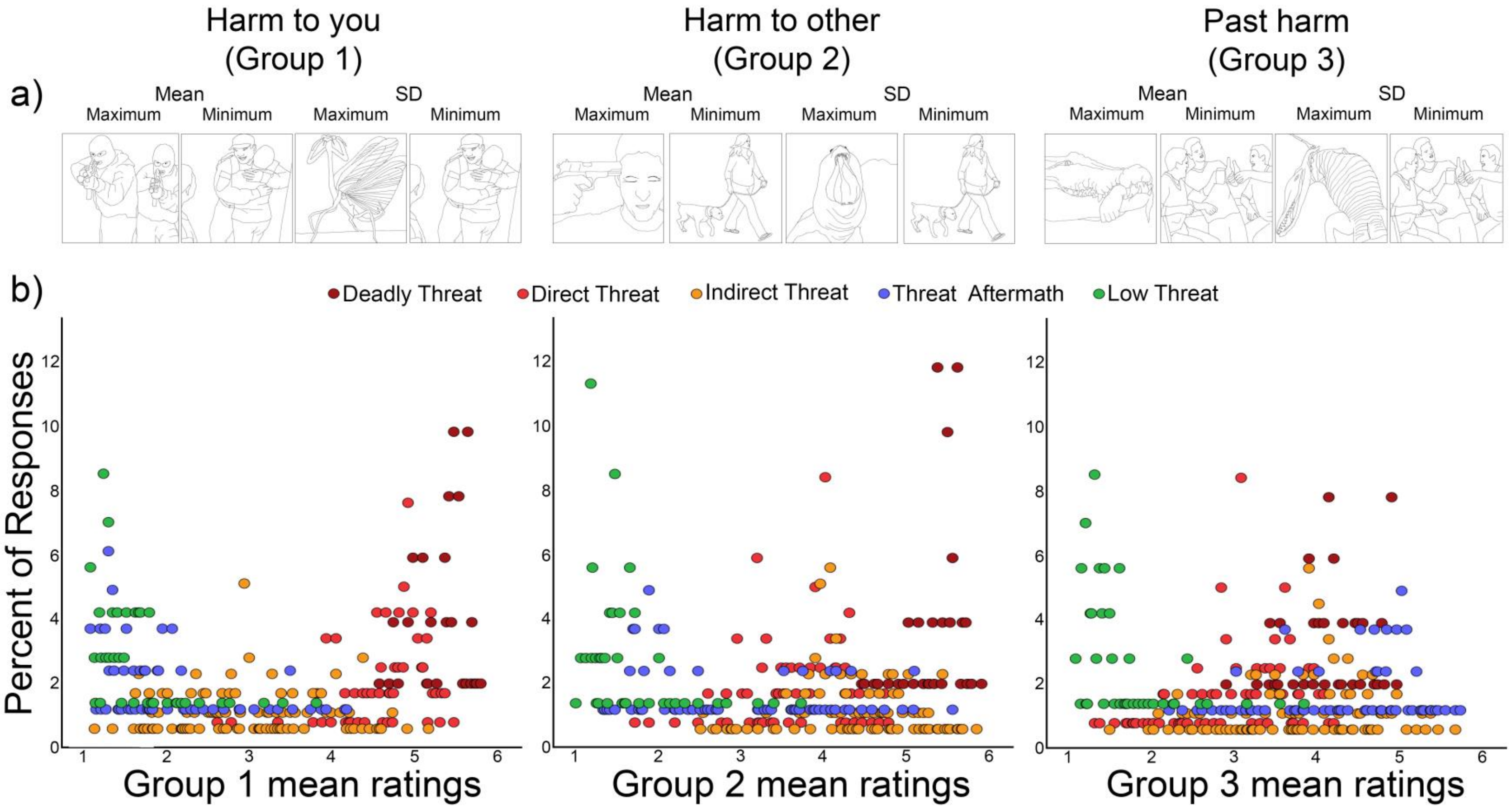

Supplemental Figure 2. Univariate distribution of image-wise mean ratings for each group (a) and image-wise standard deviations (SD) for each group (b). (c) Relationship between image-wise means and image-wise standard deviations (SD), with the best-fitting quadratic regression line for Groups 1 and 2, and the best-fitting linear regression line for Group 3.

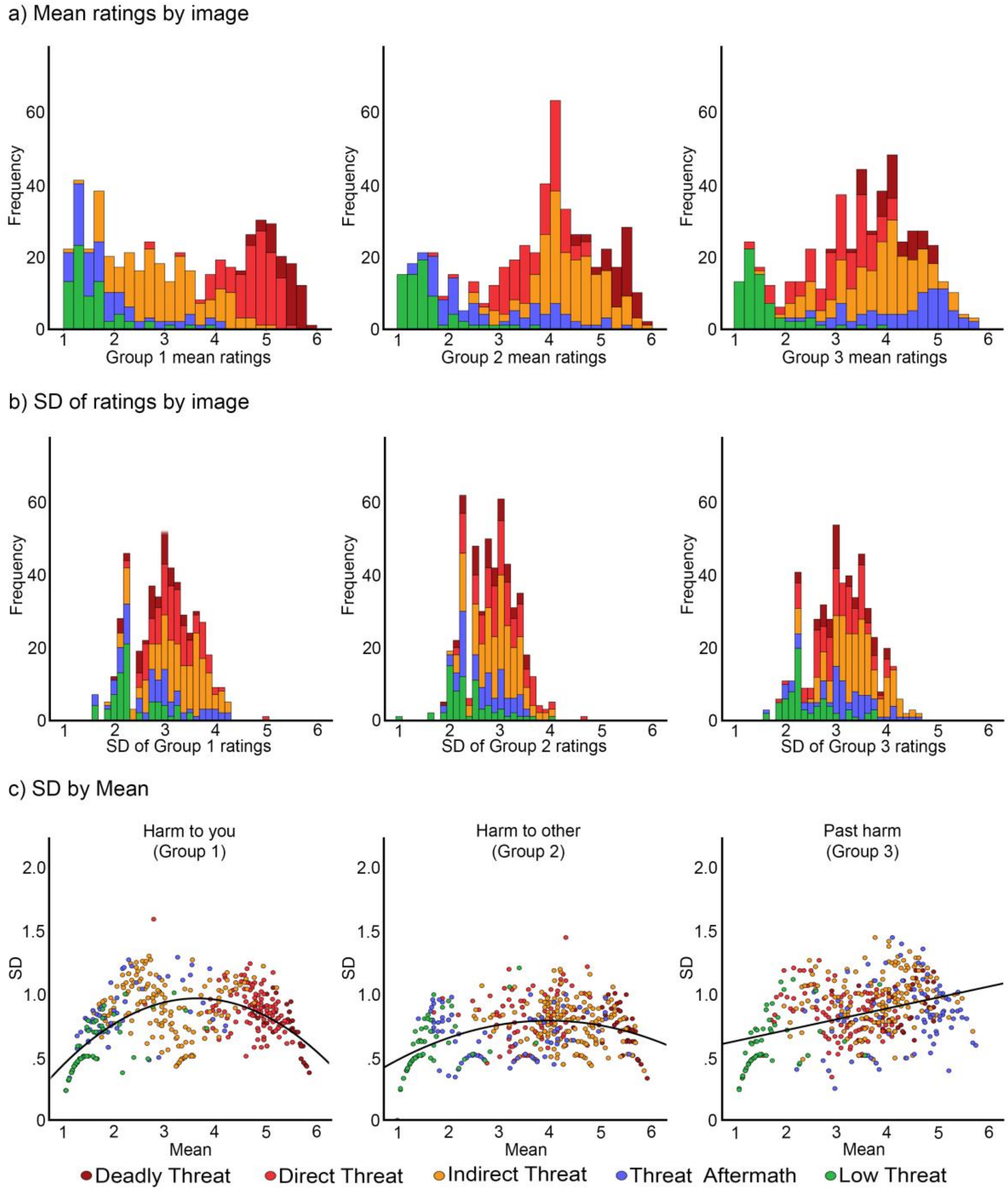

Supplement: Supplementary Material table and figures [file Supplemental_material_table_and_figures.pdf]
